# Enhancing the prediction of protein pairings between interacting families using orthology information

**DOI:** 10.1186/1471-2105-9-35

**Published:** 2008-01-23

**Authors:** Jose MG Izarzugaza, David Juan, Carles Pons, Florencio Pazos, Alfonso Valencia

**Affiliations:** 1 Structural Bioinformatics Group, Spanish National Cancer Research Centre (CNIO), C/Melchor Fernández Almagro, 3. 28029, Madrid, Spain; 2 Barcelona Supercomputing Centre, C/Jordi Girona, 29. 08034, Barcelona, Spain; 3 Computational Systems Biology Group, National Centre for Biotechnology (CNB-CSIC), C/Darwin, 3. Cantoblanco, 28049, Madrid, Spain; 4 Spanish National Bioinformatics Institute (INB), C/Melchor Fernández Almagro, 3. 28029, Madrid, Spain

## Abstract

**Background:**

It has repeatedly been shown that interacting protein families tend to have similar phylogenetic trees. These similarities can be used to predicting the mapping between two families of interacting proteins (i.e. which proteins from one family interact with which members of the other). The correct mapping will be that which maximizes the similarity between the trees. The two families may eventually comprise orthologs and paralogs, if members of the two families are present in more than one organism. This fact can be exploited to restrict the possible mappings, simply by impeding links between proteins of different organisms. We present here an algorithm to predict the mapping between families of interacting proteins which is able to incorporate information regarding orthologues, or any other assignment of proteins to "classes" that may restrict possible mappings.

**Results:**

For the first time in methods for predicting mappings, we have tested this new approach on a large number of interacting protein domains in order to statistically assess its performance. The method accurately predicts around 80% in the most favourable cases. We also analysed in detail the results of the method for a well defined case of interacting families, the sensor and kinase components of the Ntr-type two-component system, for which up to 98% of the pairings predicted by the method were correct.

**Conclusion:**

Based on the well established relationship between tree similarity and interactions we developed a method for predicting the mapping between two interacting families using genomic information alone. The program is available through a web interface.

## Background

The biological function of many proteins can only be understood in the context of their relationships with others. For this reason the biological knowledge represented in the "interactome" (the set of protein interactions for a given proteome) cannot be derived from the properties of the isolated proteins. In recent years, efforts have attempted to decipher networks of protein interactions using experimental and computational approaches [1].

One of computational techniques for predicting and studying protein interactions widely accepted by the community is that based on the similarity of phylogenetic trees. This arose from the initial qualitative observation of topological similarities between the phylogenetic trees of a number of interacting protein families [2-4]. Later these observations were systematized and quantified, and the use of tree-similarity based methods to separate interacting and non-interacting families in large sets of proteins was proposed [5,6]. This original *mirrortree *approach was subsequently improved in different ways, for example by introducing a correction for the background similarity created by the underlying speciation events [7,8]. The hypothesis that explains the relationship observed between tree similarity and interaction states that physically or functionally interacting proteins are subject to similar evolutionary pressure and forced to adapt to each other, both factors resulting in similar evolutionary histories, which are in turn reflected in similar trees. Although the co-adaptation hypothesis is still a matter of debate [9,10], the relationship between tree similarity and protein interaction has repeatedly been demonstrated by different authors in different test sets.

*Mirrortree *and related approaches indirectly measure the similarity of two phylogenetic trees by comparing the distance matrices obtained from the trees or from the multiple sequence alignments. This has been demonstrated to be a practical shortcut that avoids the complications associated with the direct comparison of trees, a problem yet to be fully resolved. To compare the distance matrices, a "mapping" or correspondence between the leaves of both trees has to be established in order to confront the appropriate elements of the matrices. Hence, having a common underlying rationale, this *mirrortree *approach has two main angles. In the first, the mapping between the sequences of the two families is known (for example implicit in the orthologous relationships when there is only one sequence in each species). In this case, the similarity of the trees according to this mapping is evaluated in order to assess the possible interaction between these two families of orthologues [5-7]. In the second, the interaction between the two families is known and the expected high similarity between their trees is used as the criteria to select the mapping (the pairings between the members of both families). This approach, introduced by Ramani & Marcotte [11] and followed by others [12-15], explores different sets of relationships between two families of interacting proteins (e.g. a family of ligands and their corresponding receptors) on the basis that the correct mapping will be that which maximizes the similarity between the trees. This situation is particularly common in eukaryotic organisms where there are many cases of large families of interacting paralogues for which only one or a few pairs of interacting proteins have been experimentally determined, and where the goal is to decipher the entire set of interactions between the two families.

The way in which most of these methods explore different mappings is by swapping pairs of columns in one of the distance matrices. It is easy to see that swapping two columns (and the corresponding rows) is equivalent to interchanging the mappings of two proteins (linking B to all proteins previously linked to A and vice versa). The exhaustive exploration of all possible mappings will require *n! *calculations for a set of *n *elements, which soon becomes unfeasible for large protein families. For this reason, current methods use heuristic approaches (i.e. Monte Carlo) to perform a "guided" non-exhaustive exploration of the solution space. Due to their intrinsic heuristic nature, these methods do not ensure the global best solution will be found, and they are easily trapped within local sub-optimal solutions. To partially overcome this problem, these methods are run several times in the search for a consensus between the different analyses, commencing each execution from a different point in the solution space. Some of the methods also make use of the fact that the distance matrices have an underlying tree structure behind them that imposes some restrictions on the possible mappings, thereby reducing the search space [15].

Another intuitive way of decreasing the number of mappings to be explored is by avoiding pairings between proteins of incompatible "classes", for example between proteins of different species in families that contain both orthologues and paralogues. We have developed a new Monte Carlo based method to predict the mapping between two interacting protein families. This method overcomes some of the limitations of previous approaches by taking advantage of the information available on the species from which the trees are derived. Basically, the method does not allow pairings involving proteins from different species, or "incompatible classes" in general. This is different to applying the previous equivalent approaches separately to each of the species (or classes), since even if the mapping is restricted to intra-organism pairings the information of the whole tree is used to assess these mappings.

We evaluated the performance of this method on a large set of interacting protein domains and a well described case of co-evolving interacting families: the sensor kinases and response regulators of the Ntr-type two component system. This analysis proved to be more accurate than previous approaches. Additionally, the spectrum of variables related to the number of proteins within the families, the number of species in the alignments, the complexity of the trees, etc., could for the first time be explored to set the boundaries of the expected accuracy and its dependence on the characteristics of the data.

## Results and Discussion

We first present the results for the large dataset of interacting domains. This allows to obtain performance figures based on a statistically large number of examples. Then, we discuss in detail the results for the Ntr-type two-component system.

### Large-scale evaluation based on interacting protein domains

**Figure 1:**
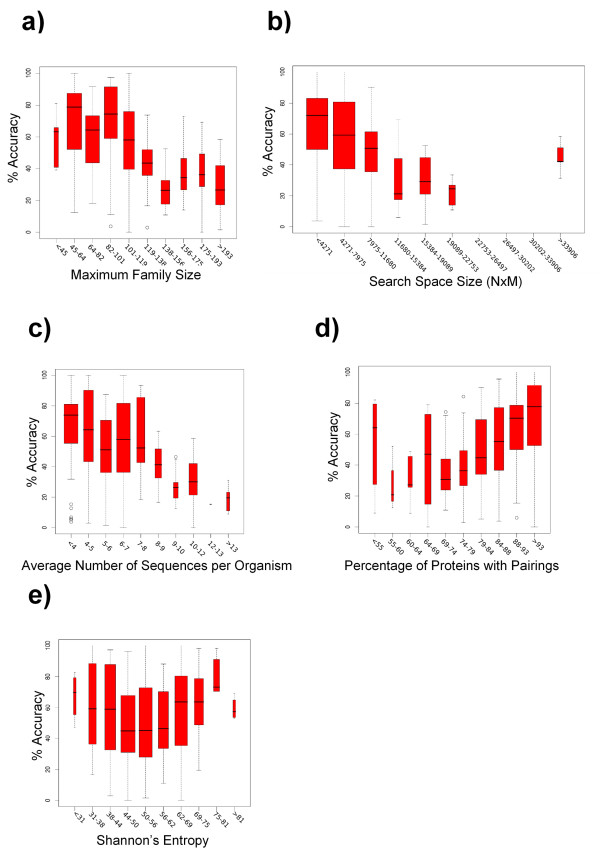
**Dependence of the accuracy on the characteristics of the input data for the large scale test set of protein domains**. The results are shown as boxplots [16]representing the lower quartile, upper quartile, median, largest observation and outliers for each of the classes.

Figure 1 represents the accuracies obtained for the predicted mappings in relation to different characteristics of these domains. Only the results for the distances based on the percentage divergence (see Methods) are shown. The other two measures of distance produced very similar results (Additional file 1, Figure S1). The boxplots [16] representing the lower quartile, upper quartile, median, largest observation and outliers, show that the accuracy of the predictions varies significantly in the set of domain pairs and that it is highly dependent on the characteristics of the corresponding families/alignments (see below). The average accuracy for the whole dataset is 55.5%. For 56% of the cases the accuracy is higher than 50%, and for 27% the accuracy is higher than 75%. For the most populated classes (the widest bars in the boxplots) the accuracies were around 60–80%.

The relationship between accuracy (percentage of correct pairings) and family size (number of proteins in the largest family of the pair) shows that smaller families tend to produce better results (Figure 1a). This makes sense since the smaller the family the smaller the number of possible mappings (size of the solution space) and hence, it is easier for the algorithm to find a good mapping. For most of the proteins (most populated classes, between 45 and 100 members in the largest family) the accuracy values are around 50–80%.

Figure 1b shows the dependence of the accuracy on the size of the search space (NxM, N and M being the sizes of the two families). As expected the accuracy decreases with the size of the space of solutions, since it is more difficult for the algorithm to find the right solution. Nevertheless, this plot also shows that most pairs correspond to relatively small search spaces (widest bars) and hence they produce reasonable accuracy values, around 60%.

In terms of the relationship between the accuracy and the average number of paralogues (sequences per organism), the method performs better when there are fewer paralogues for each organism (Figure 1c). As the number of paralogues decreases, the size of the solution space also decreased drastically, since only pairs between proteins from the same organism were admitted.

The relationship between the accuracy and the number of pairings between the two families, that is the percentage of proteins that were involved in an interaction (represented as the percentage with respect to the number of proteins in the smallest family) was assessed (Figure 1d). It must be remembered that for the test set to remain as close to the real situation as possible, we did not require all the proteins to be paired. Indeed, the test set contains pairs of domains with many members in the corresponding families for which only a few were involved in interactions. It can be seen that it is easier for the method to succeed when more proteins are involved in interactions, while the results are worse when a smaller fraction of proteins were involved in interactions.

The dependence of the accuracy on the information content of the trees is shown in Figure 1e. It is clear that this method works better as the trees contain more information. Trees with low information content have a characteristic "star-like" shape, meaning that all the distances between leaves are very similar. This similarity of the distances makes ambiguous the matching with other trees, since there are many equivalent possibilities producing the same score. So, in general trees with high information content are associated to better results. However, it can be seen that trees with very low information content (low entropy) also produce good results. This is an artefact due to the fact that the entropy is not totally independent on the number of sequences. Trees with few sequences tend to produce low entropies and good results since the size of the search space is smaller (Figure 1a, discussed above). If we disregard this artefact it was clear that more informative trees produced better results.

**Figure 2:**
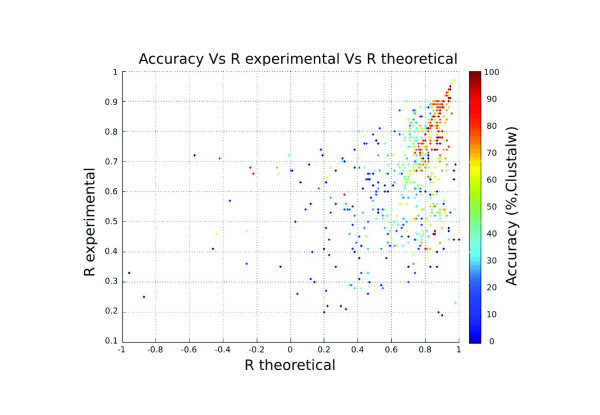
**Dependence of the accuracy on the similarity of the trees**. The similarity between the trees of the two interacting families is quantified as the Pearson's correlation coefficient. Both the similarity of the trees according to the real mapping ("R theoretical", which may or may not be found by the heuristic method) and the similarity according to the best mapping found by the method ("R experimental") are represented. The accuracy is shown in a colour scale.

The whole methodology is based on the fact that interacting families show similar phylogenetic trees. Even if this is true for most cases, it has been shown that the trees of some particular interacting families are not correlated [6,7]. In these cases, TAG_TSEMA, or any other method based on tree-correlations, would not be expected to work. To quantify this, we studied the relationship between accuracy and tree similarity for both the real mapping and for the best solution found by the heuristic method (Figure 2). It can be seen that there is a clear relationship between both similarities and the accuracy, although this relationship was higher for the similarity obtained from the real mapping. Hence, the method would be expected to work well for pairs of families with a clear co-evolutionary behaviour, as represented by a high similarity of their corresponding phylogenetic trees.

Trying to better evaluate the contribution of "tagging" to the improvement of the results, we represent the relationship between the accuracy, the average number of sequences per tag (organism) and the number of organisms. If tagging is improving the results, the highest accuracies should be in the region of large number of organisms and low sequences/organism. This seems to be the case (Additional file 1, Figure S3).

**Figure 3:**
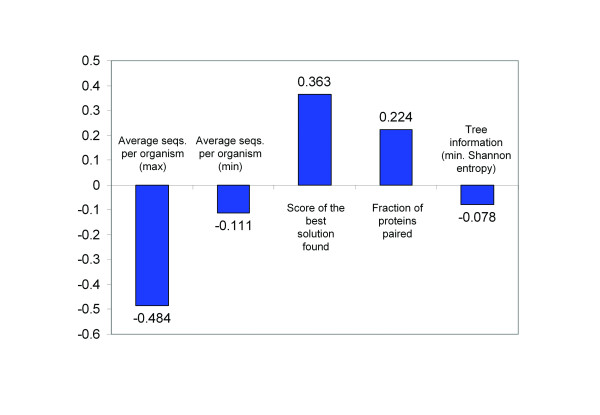
**Coefficients of the multiple regression**. The coefficients of the variables for the multiple regression, representing the dependence between the accuracy and these parameters, are shown. The absolute value of these coefficients represents the relative importance of each of the parameters in determining the accuracy. Their sign represents whether they contribute positively or negatively to this accuracy.

In order to compare the influence of all the different factors mentioned above on the performance of the method, we performed a multiple regression in which the independent variables were these factors and the dependent variable was the accuracy. The corrected R^2 ^of this multiple regression is 0.504, indicating that the success (accuracy) is clearly dependent on these factors. The coefficients of the different factors in the regression indicate the extent to which each of them contribute to the accuracy, and whether this contribution is positive or negative (Figure 3). The most influential factor is the average number sequences per organism, and the negative sign indicates that it contributes negatively (as seen in Figure 1c). The next factors in terms of their importance in determining accuracy, are the score for the best solution found by the algorithm and the fraction of proteins that have some pairing. Apparently, the accuracy is relatively independent on the information content of the trees, as reflected in the coefficient close to 0. However, this is an artefact due to the "U" shape of the boxplot representing the relationship between the entropy and the accuracy discussed previously (Figure 1e). This shape brought the global best fit line closer to the horizontal, which is indicative of no dependence.

### Ntr-type two-components system

**Figure 4:**
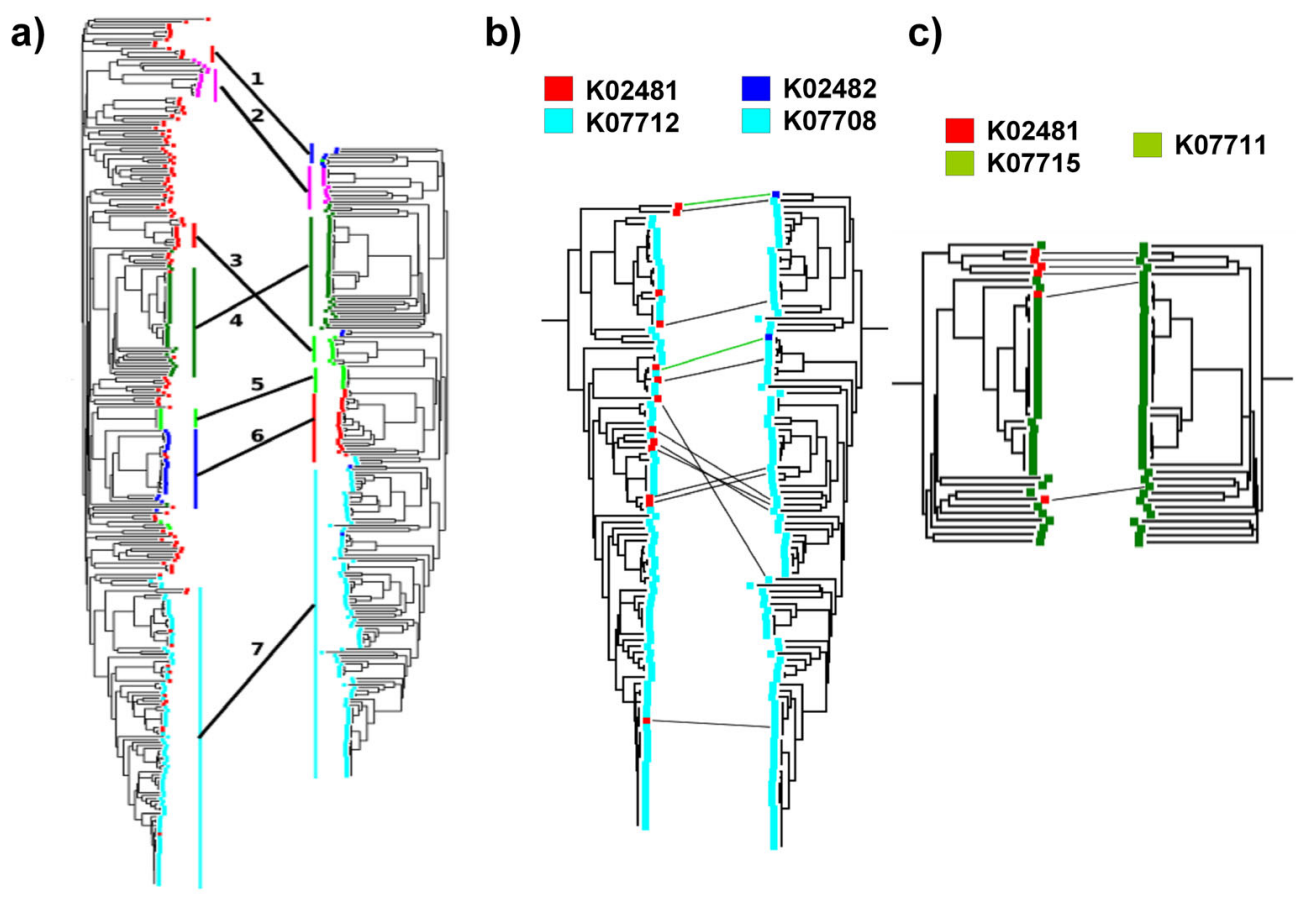
**Mapping between the sensor kinase and the response regulator families of the Ntr-type two-component system**. In the pairs of confronted trees, the tree on the left corresponds to the response regulators and the one on the right to the sensor kinases. The representations of the trees were generated with MEGA [21]. a) Schematic representation of the predicted pairings between KOs. Within each of the trees, the colour of the leaves corresponds to the 6 KO (12 in the two trees). The inter-tree lines represent the predicted KO pairings, derived from the individual pairings between proteins. These KO pairings are in full agreement with the KO-KO interactions based on the original 14 pairs of interacting proteins (see Materials and Methods). b and c) Detailed view of interactions #4 and #7 in panel *a *and the corresponding sub-trees. In these panels, the inter-tree black lines represent the potentially incorrect protein pairings predicted by the method. In these two examples, it can be seen that all these potentially incorrect pairings are possibly due to wrong KO assignments (see Results). The two green lines represent correct pairings even though the KO assignments look incorrect. Figure S2 (supplementary material) shows all the pairs in the predicted mapping, and Table S2 contains the whole list of protein pairs within that mapping indicating whether they are correct or not.

To illustrate the predictive power of this method in a real-life scenario, we tested the method with a well-studied example of two interacting protein families: the sensor kinases and transcriptional regulators of the Ntr-type two-component system. Using these families, Ramani and Marcotte successfully predicted 57% of the correct protein pairs and up to 86% of the correct KO pairs [11]. Figure 4a shows an overview of the mapping predicted by TAG-TSEMA for these two families. Additionally, a figure showing the whole mapping and a table with the list of pairings within this mapping, indicating the correct ones are available as supplementary material (Additional file 1, Figure S2 and Table S2). The best mapping found comprises 205 pairings between the two families, 88% of which are correct according with the KO assignments. From the original 14 pairings described in the literature (see Methods), our method correctly predicted 13 (93%). Nevertheless, there are some cases where the KO assignments are possibly incorrect. Analysing the detailed structures of the trees, it was clear that some proteins were wrongly assigned to a given KO by the automatic method used in KEGG (Figures 4b and 4c). For example, the proteins in the right tree of Figure 4b (response regulators) assigned to the dark-blue KO (KO2482) should in reality be assigned to the light-blue KO (KO7708) note the positions of these dark-blue proteins within the light-blue clade, their distances to light-blue proteins, and the relative positions of the light-blue and dark-blue KO in the trees of Figure 4a). Similarly, another case of a possible wrong assignment is shown in Figure 4c. When these potential errors in KO assignments are manually corrected (see Table S2 for a complete list), the accuracy rises to 98% (from the original 88%) and, in fact, most of the wrong pairings (i.e. lines in Figures 4b and 4c) were due to these incorrect KO assignments. Only 4 of the 205 pairs are still incorrect after this manual correction of KO assignments (Table S2). Even more, 1 of these 4 wrong pairings could be explained by the fact that it involves an organism for which only 1 protein is present in each one of the trees, a situation whereby the automatic method has no other choice than to link them.

For comparative purposes, we run this method using RMSD as scoring function and obtained an accuracy of 86% pairs correctly predicted. Comparing with the corresponding figures for Marcotte's published data (57%, which also uses RMSD as distance measure), and this method with Pearson/Student-t (93%), it looks like the improvement comes from both, the usage of Pearson correlation, and the "tagging", and that this last factor produces a significant increase in the accuracy. To further asses the individual effect of the "tagging" in the accuracy, we ran the pairs of trees for each organism separately. In this case we used those organisms with at least three proteins in both families (22 organisms). With this procedure, we could retrieve 21 correctly assigned K0 pairings out of 77 (27,3%), an accuracy which is quite low compared to the results using tagging (see above). This shows how predicting a mapping using tag restrictions is better than running each one of the tags (organisms) separately and joining the results afterwards. The reason could be related to the inclusion of more information for discriminating mappings (inter-organism distances) while not increasing the size of the search space (due to the tag restrictions). Nevertheless, all these results should be taken carefully since they are based on a single example and a deeper analysis would be required to better dissociate the contribution of these factors.

## Conclusion

In this work, we present a method for the prediction of the mapping between two families of interacting proteins based on the well-established relationship between similarity of phylogenetic trees and protein interactions. The method is able to incorporate information on the belonging of proteins to classes in order to restrict the protein-protein pairings according to these classes. The most general class we can define is the organism that the proteins belong to, in this way avoiding "heterologous" interactions between proteins from different organisms. Many other classes can also be defined, such as the cellular compartment, co-expression, etc. We show that these restrictions improve the performance of the method. It is important to note that applying this method to an example with N classes (i.e. N organisms) is different from applying other similar approaches N times to N pairs of trees, each one containing only paralogues for a given organisms. This fact, illustrated for the NtrC example, is possibly related to the inclusion of more information for distinguishing mappings (inter organism distances) while keeping the search space relatively small due to the restrictions associated to the classes.

Moreover, for the first time we apply here a tree-similarity-based method to predict pairings in a large set of interacting domains, which allowed us to obtain statistically significant figures regarding performance, and to asses their dependence on the characteristics of the input data. Previous studies have only tested the methodology employed on a very limited number of examples. Although this dataset is "unrealistic" in the sense that it is composed of protein domains instead of complete proteins, we have tried to keep the level difficulty as realistic as possible. For example, unpaired domains were left in the alignments that obviously makes it harder for the method to find the right solution. It is important to note that in our experience, this is the only large-scale test set possible, since there is little systematic information available on the interaction between members of proteins families that can be used to construct a large dataset of full-length proteins. Indeed, this lack of information strengthens the need for methods like that presented in this work. We complemented this large-scale set with a more "real-life" example that involves a well-described case of interacting families. In this case the method produces substantially better results than previous similar ones.

There are large families of interacting proteins for which only one or a limited number of pairs of interacting proteins have been experimentally determined (i.e. Ras/Ras effectors, chemokines/chemokine receptors). In many of these cases, which are highly significant in eukaryotic organisms, the differential interaction of the members of the two families is crucial to explain their biological roles. It is expected that with the continuous stream of genomic sequences, we will see more and more of these examples. Thus, it is in this context that the automatic method presented here can provide clues about specific interactions, complementing other computational and experimental techniques in the search for the molecular basis of functional specificity.

## Methods

### Description of the Algorithm

TAG-TSEMA uses a Monte Carlo algorithm to perform a directed non-exhaustive exploration of the solution space (possible mappings). Proteins can be "tagged" according to their membership to classes, and pairings (interactions) are permitted between proteins belonging to the same class while inter-class pairings are forbidden. In this work, we label each protein according to the organism it belongs to, restricting the possible pairings to those involving proteins from the same organism. It is important to note that this restriction of the search space through the creation of classes with external information permits many other possibilities, such as tissue-specific expression (disregarding pairings between proteins from different tissues), sub-cellular localization, temporal co-expression, etc. The method can also deal with an additional class, called *undef*, for proteins that were not associated to any given class.

For the examples discussed here in which the classes correspond to organisms, proteins with the same tag (within each one of the trees) are paralogues. During the heuristic search for an optimal solution, only pairs of proteins of the same organism (or involving proteins with the *undef *tag) are allowed. Apart from its obvious biological rationale this restriction has two main advantages:

1) The computational complexity is reduced from *n! *(where *n *is the number of members in the family) to *Σn*_*i*_*! *Where *n*_*i *_is the number of proteins within a given class, being the sum-up done along the whole set of classes (organisms). Given that the number of elements in the distance matrix is the main constraint for the feasibility of the calculation, by reducing the size of the search space the approach proposed here can deal with larger protein families.

2) By including paralogue/orthologue relationships, we are reinforcing the protein interaction signal between given protein pairs for each organism. This is because we are adding more parallel links between sets of interacting orthologues, while avoiding the increase in the complexity of the problem due to artificial inter-species coupling.

The pseudo-code for TAG-TSEMA algorithm could be summarized as follows:

1. Generate the phylogenetic trees for both families starting from their multiple sequence alignments. Different tree-construction methods have been used in this work for the purpose of comparison.

2. Build the corresponding distance matrices, calculating the distances by summing the lengths of the branches separating each pair of nodes in the trees.

3. Randomly shuffle one of the matrices to establish a set of initial (tag-restricted) random mappings (1000 by default). Each of these random mappings represents a different starting point in the solution space where the Monte Carlo searches can start.

4. For each one of these initial random mappings.

4.1. Calculate the starting correlation between the two distance matrices according to the mapping.

4.2. Randomly select a class (organism) and a protein within this class in the second matrix to be swapped with another feasible protein (*undef *or of the same class). The rows and columns corresponding to these two proteins are swapped.

4.3. Re-calculate the correlation between the distance matrices according to the new mapping obtained after the swap. If the correlation is better the change is accepted and if not, the previous mapping is recovered (that before row swapping).

4.4. Repeat a predefined number times from step 4.2 (1 million by default) and retain the best mapping found (that with the highest correlation).

5. The overall best solution obtained for all the initial mappings is recorded, together with the best solutions of each of the mappings.

The "cooling temperature" parameter of the Monte Carlo algorithm is set to 0.95.

The algorithm implemented in C includes the possibility of confronting two families with a different number of proteins, which is the situation for most real interacting families, due to promiscuity, pseudogenes, etc. In the current implementation of the algorithm it is also possible to use three alternative scoring functions to assess the correlation between the distance matrices and hence, the proposed mappings: root mean square deviation (RMSD), Pearson's linear correlation, and its corresponding Student's *t*. However, in order to compare families of very different sizes, all the results in the forthcoming analysis are based on the Student's *t *test. We used the value of the Student's *t *itself, instead of the associated p-values.

As a measure of tree-complexity we use the Shannon entropy of the distribution of tree distances, after binning them in classes of the same width. Thus, the entropy of a given tree is defined as:

$$H=-\sum_{i=1}^{N}p_{i}\cdot \log_{2}p_{i}$$

Where the sum runs for all the distance bins (N = 15 in this work), and *p*_*i *_is the fraction of distances within the *i*^*th *^bin.

### Test sets

We tested the method with a well-studied case of two interacting protein families, allowing us to compare it with other equivalent methods which used the same example. Additionally, we also assessed the performance of the method on a large dataset of interacting protein domains which, despite being a little far from a real-world application, has many of the characteristics of full-length proteins. This large dataset allows us to obtain statistical information regarding the performance of the method. It would have been desirable to test the method with a large dataset of whole-length proteins that contained a reasonable amount of information on the interactions between their members. However, such a dataset does not currently exist and, in fact, the lack of information on interactions between family members that would be necessary to build such a dataset highlights the importance of methods like that presented here.

### Large scale dataset of interacting protein domains

We tested the new method on a large set of 604 protein domains (forming 488 interacting pairs) for which the right mapping is simply given by the presence of the two domains in the same protein chain. Due to its large size, this set allows a statistical assessment of the performance of the method and its dependence on the characteristics of the data.

We started by retrieving pairs of domains from Pfam [17] that could be linked to different regions of the same *Yeast *protein (*i.e.: *different regions of the same protein are asociated to the two domains). For example, if a Yeast protein *A *matches two Pfam domains (*X *and *Y*), in its N-terminal and C-terminal regions respectively, we would take *X-Y *as a pair of interacting Pfam domains. For this pair of interacting Pfam domains, the "real mapping" (set of links between the actual sequences within these Pfam domains) is given by the co-presence of these sequences in the same protein chain. I.e. An homolog of protein *A *(*A'*) associated to the same domains *X *and *Y*, would define a link (between the sequences of its N-terminal and C-terminal parts). Protein *A *itself would also define another link. All the links between *X *and *Y *constitute the "real mapping", which is compared with the mapping predicted by the method for this particular pair *X-Y*.

In order to be sure about the uniqueness and quality of the sequences, we used only eukaryotic proteins that have an entry in SwissProt (hence removing those coming from TREMBL). Then, we filtered out those low quality sequences annotated as "fragment", "hypothetic" or "putative" proteins. Finally, we removed those pairs of domains with less than fifteen sequences mapped between them. We also removed pairs of domains in which any of the two members involved less than four organisms with more than 3 sequences each, and the ones with less than 50% of the proteins paired (with respect to the family with the lowest number of sequences). This process yielded 488 interacting pairs of domains, comprising 604 individual domains.

It is important to note that this set represents an especially tough test for the method, because not all the sequences have to be paired for a given pair of domains. This is similar to an extreme real case in which we have information about the interaction between two proteins, but no clue about the extension of their families. Therefore, our set is full of cases with few domain-domain interactions that we would expect to produce poor predictions using our approach (poor co-evolutionary signals with a lot of noise). We did not impose this or any other restrictions in order for the cases to be as close to the real situation as possible.

For each domain within this test set, we align its sequences with MUSCLE [18]. In order to test the influence of different estimators of protein divergence, we generated three sets of phylogenetic trees using the Neighbour Joining algorithm with three different evolutionary distances: simple percentage of divergence without including gaps (as implemented in ClustalW [19]: (100-%id)/100, where %id is the percentage of identical residues), Kimura's correction, and Scoredist (both as implemented in *Belvu*, E. Sonnhammer, unpublished). Given the large-scale nature of this test we were limited to the Neighbour Joining algorithm for the generation of the trees. However, we would expect that more accurate trees will improve the results of our method.

Although the program can be run on any computer, its application to this large set of examples requires a lot of computer power. Hence, that test set was run on MareNostrum (MN), one of the largest computers in the world dedicated to science. For this specific task, approximately 730 hours of MN computer time were used to run TAG-TSEMA for these 488 pairs of domains, distributed over 32 processors, with an average of 10 minutes for each pair. The execution of a single pair comprises 500 complete runs of the entire Monte Carlo search consisting of a million steps each.

For each one of the 488 pairs of domains in the test set, the accuracy (percentage of correct pairings respect to the real mapping-see above-) of the top-scoring solution was calculated.

### Ntr-type two-components system

We also tested the method with a more realistic case of two interacting families of full-length proteins for which enough information on the pairings between their members is available. The interactions between the Sensor kinases and the corresponding Response Regulator proteins in the Ntr-type two-component system has been previously studied in by Ramani and Marcotte [11], and we followed a similar strategy to construct this test set. We retrieved the 14 documented interactions between individual response regulator and kinases from KEGG [20]. According to the KEGG orthology assignments, both the 14 sensor kinases and the 14 response regulators are classified into 6 sets of KEGG's functional orthologues (KOs). Hence, we established the corresponding KO-KO interactions based on the original 14 protein-protein interactions (Additional file 1, Table S1), a process that results in 7 KO-KO interactions. To build the entire families we retrieved all the sequences present in KEGG for these 12 KOs (6 from each tree), and generated the MSAs and the trees by using the approach previously described for the protein domains dataset. By following this procedure we obtained a tree comprised of 213 proteins for the sensor kinase family and another with 286 proteins for the response regulator family. We can evaluate the mapping between these two trees predicted by TAG_TSEMA either based on the original 14 documented interactions, or through the KO-KO interactions extrapolated from them.

## Availability and Requirements

The program TAG-TSEMA can be used through the following web server: . This server combines the method described here with an interactive visualization and post-processing of the results as described in [14]. In this server, the assignment of the proteins to classes (organisms, subcellular localization, etc) is indicated in the input files with the "_" symbol, using a Swissprot-like nomenclature (i.e. "prot1_class1", "prot2_class2", etc).

## Authors' contributions

JMGI and DJ generated the initial idea and prepared the datasets. JMGI implemented most of the software. CP adapted the software and performed the runs in the supercomputer. JMGI, DJ and FP analyzed the results. FP, DJ and AV wrote the paper. All authors read and approved the final manuscript.

## Supplementary Material

Additional File 1 Supplementary tables S1 and S2 and supplementary figures S1, S2 and S3 Click here for file

## References

[B1] Uetz P, Finley RL (2005). From protein networks to biological systems. FEBS Lett.

[B2] Fryxell KJ (1996). The coevolution of gene family trees. Trends Genet.

[B3] van Kesteren RE, Tensen CP, Smit AB, van Minnen J, Kolakowski LF, Meyerhof W, Richter D, van Heerikhuizen H, Vreugdenhil E, Geraerts WP (1996). Co-evolution of ligand-receptor pairs in the vasopressin/oxytocin superfamily of bioactive peptides. J Biol Chem.

[B4] Pages S, Belaich A, Belaich JP, Morag E, Lamed R, Shoham Y, Bayer EA (1997). Species-specificity of the cohesin-dockerin interaction between Clostridium thermocellum and Clostridium cellulolyticum: prediction of specificity determinants of the dockerin domain. Proteins.

[B5] Goh C-S, Bogan AA, Joachimiak M, Walther D, Cohen FE (2000). Co-evolution of Proteins with their Interaction Partners. J Mol Biol.

[B6] Pazos F, Valencia A (2001). Similarity of phylogenetic trees as indicator of protein-protein interaction. Protein Eng.

[B7] Pazos F, Ranea JAG, Juan D, Sternberg MJE (2005). Assessing Protein Co-evolution in the Context of the Tree of Life Assists in the Prediction of the Interactome. J Mol Biol.

[B8] Sato T, Yamanishi Y, Kanehisa M, Toh H (2005). The inference of protein-protein interactions by co-evolutionary analysis is improved by excluding the information about the phylogenetic relationships. Bioinformatics.

[B9] Hakes L, Lovell S, Oliver SG, Robertson DL (2007). Specificity in protein interactions and its relationship with sequence diversity and coevolution. Proc Natl Acad Sci USA.

[B10] Mintseris J, Weng Z (2005). Structure, function, and evolution of transient and obligate protein-protein interactions. Proc Natl Acad Sci USA.

[B11] Ramani AK, Marcotte EM (2003). Exploiting the co-evolution of interacting proteins to discover interaction specificity. J Mol Biol.

[B12] Tillier ER, Biro L, Li G, Tillo D (2006). Codep: maximizing co-evolutionary interdependencies to discover interacting proteins. Proteins.

[B13] Gertz J, Elfond G, Shustrova A, Weisinger M, Pellegrini M, Cokus S, Rothschild B (2003). Inferring protein interactions from phylogenetic distance matrices. Bioinformatics.

[B14] Izarzugaza JM, Juan D, Pons C, Ranea JA, Valencia A, Pazos F (2006). TSEMA: interactive prediction of protein pairings between interacting families. Nucleic Acids Res.

[B15] Jothi R, Kann MG, Przytycka TM (2005). Predicting protein-protein interaction by searching evolutionary tree automorphism space. Bioinformatics.

[B16] Tukey J (1977). Exploratory Data Analysis.

[B17] Bateman A, Coin L, Durbin R, Finn RD, Hollich V, Griffiths-Jones S, Khanna A, Marshall M, Moxon S, Sonnhammer EL, Studholme DJ, Yeats C, Eddy SR (2004). The Pfam protein families database. Nucleic Acids Res.

[B18] Edgar RC (2004). MUSCLE: multiple sequence alignment with high accuracy and high throughput. Nucleic Acids Res.

[B19] Chenna R, Sugawara H, Koike T, Lopez R, Gibson TJ, Higgins DG, Thompson JD (2003). Multiple sequence alignment with the Clustal series of programs. Nucleic Acids Res.

[B20] Kanehisa M, Goto S, Kawashima S, Okuno Y, Hattori M (2004). The KEGG resource for deciphering the genome. Nucleic Acids Res.

[B21] Tamura K, Dudley J, Nei M, Kumar S (2007). MEGA4: Molecular evolutionary genetics analysis (MEGA) software version 4.0. Mol Biol Evol.

