# Supplementary material for: Enhancing the prediction of protein pairings between interacting families using orthology information
Source: BMC Bioinformatics. 2008 Jan 23;9:35. doi: 10.1186/1471-2105-9-35 (PMC2263026; doi:10.1186/1471-2105-9-35)

Enhancing the prediction of protein pairings between interacting families using orthology information

Supplementary Material

Jose M. G. Izarzugaza, David Juan, Carles Pons, Florencio Pazos[*], Alfonso Valencia

*Corresponding author
pazos@cnb.uam.es
Tlf. +34.915854669
Fax. +34.915854506

Table S1. List of the 14 experimentally described links between members of the sensor kinase and response regulator families of the Ntr-like two component system. The seven links between KEGG orthologous (KO) groups implicitly produced by these 14 links are shown below. All the members of these linked KOs are assumed to interact.

**Organism**	**Protein_1**	**KOG_1**	**Protein_2**	**KOG_2**
Eco	B3869	K07708	B3868	K07712
Eco	B4003	K07709	B4004	K07713
Eco	B2219	K07710	B2220	K07714
Eco	B2556	K07711	B2554	K07715
Sty	STY3875	K07708	STY3876	K07712
Sty	STY2634	K08475	STY2633	K08476
Sty	STY3712	K07709	STY3711	K07713
Hpy	HP0244	K07710	HP0703	K02481
Hpj	JHP0229	K02482	JHP0643	K02481
Ctr	CT467	K07710	CT468	K02481
Cpn	CPN0584	K07710	CPN0586	K02481
Bbu	BB0764	K02482	BB0763	K02481
Aae	Aq_1115	K02482	Aq_1117	K02481
Aae	Aq_231	K02482	Aq_230	K02481

```
1.- K02481 - K02482
2.- K08476 - K08475
3.- K02481 - K07710
4.- K07715 - K07711
5.- K07714 - K07710
6.- K07713 - K07709
7.- K07712 - K07708
```

Figure S1. Boxplots showing the relationship between the accuracy and different characteristics of the pairs of domains for trees based on *Kimura* and *Scoredist* distances. See main text for details.

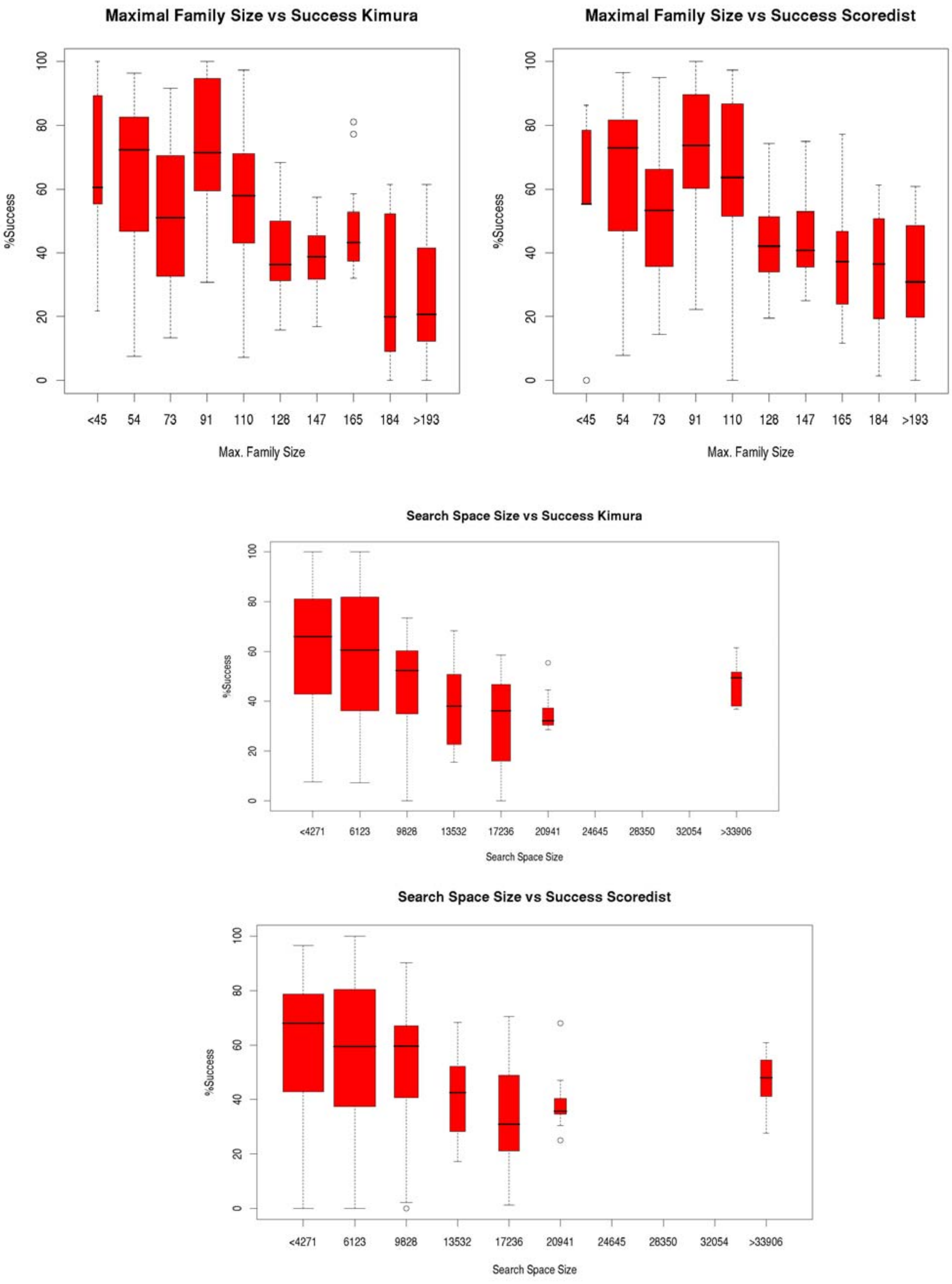

Figure S1 (cont.)

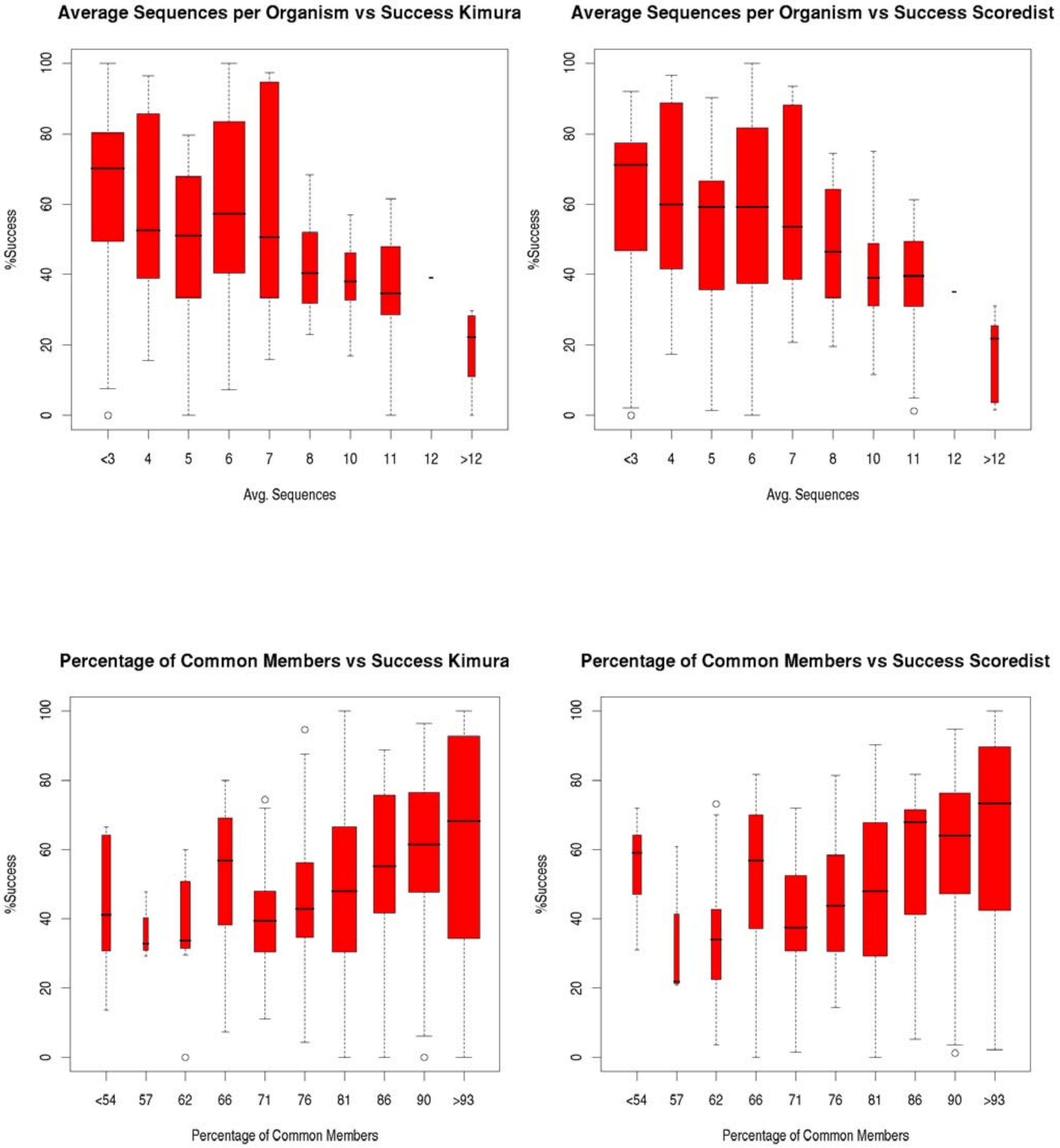

Figure S1 (cont.)

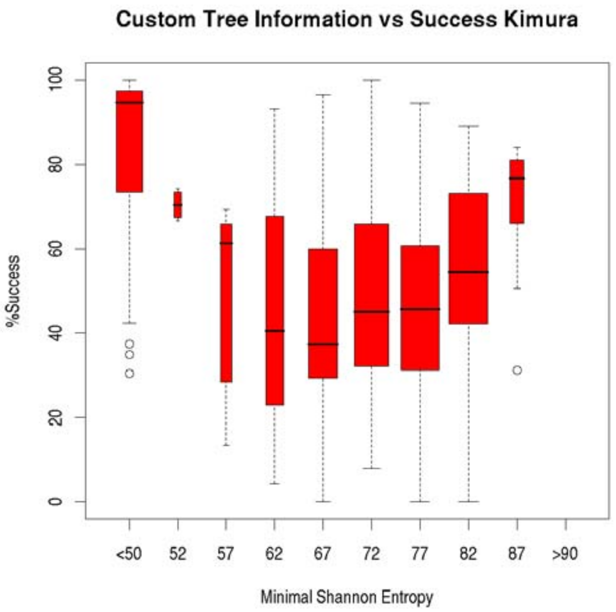

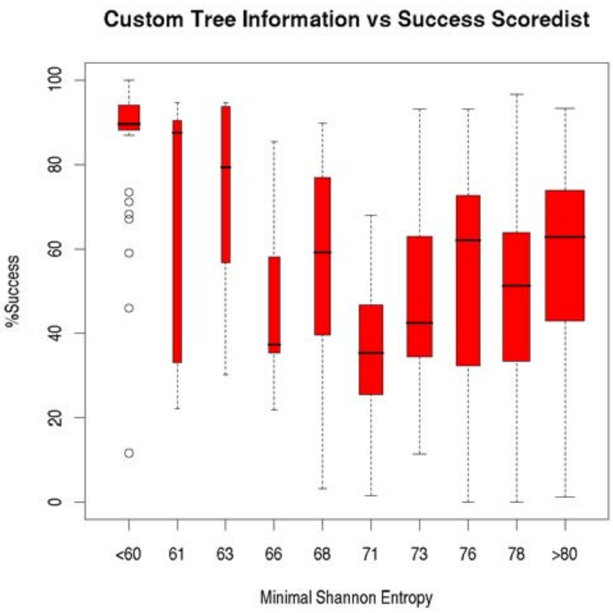

Table S2. Predicted pairings between the sensor kinase and response regulator families of the Ntr-like two component system corresponding to the best mapping found by TAG-TSEMA. The "reliability" and "disgregation" scores for each pairing are shown (see Izarzugaza *et al.* (2006). Nucleic Acids Res., 34:W315-319). The last column indicates whether that particular pairing is correct ("**OK**"), clearly wrong ("**x**") or wrong possibly due to a wrong KO assignment ("bad KO (?)"). See the main text for details. A graphical representation of this mapping is shown in Figure S2.

SENSOR	KO	RESPONSE	KO	RELIABILITY	DISGREGATION	HIT?
tcx:Tcr_0905	K07708	tcx:Tcr_0906	K07712	98.4	96.8	OK
rpc:RPC_2577	K07708	rpc:RPC_2578	K07712	100	100	OK
ypa:YPA_1084	K07709	ypa:YPA_1085	K07713	74.8	50.6	OK
noc:Noc_0783	K07711	noc:Noc_0785	K02481	73.2	46.4	bad KO (?)
ilo:IL2436	K07708	ilo:IL2435	K02481	100	100	bad KO (?)
plu:plu0236	K07708	plu:plu0235	K07712	97.4	94.8	OK
ecc:c3079	K07711	ecc:c3077	K07715	73.2	48	OK
eci:UTI89_C4458	K07708	eci:UTI89_C4457	K07712	97.4	94.8	OK
pcu:pc1365	K07710	pcu:pc1364	K02481	100	100	OK
sfx:S2775	K07711	sfx:S2773	K07715	73.2	48	OK
stm:STM2397	K08475	stm:STM2396	K08476	100	100	OK
pst:PSPTO_0353	K07708	pst:PSPTO_0352	K07712	100	100	OK
bms:BR1118	K07708	bms:BR1117	K07712	100	100	OK
ret:RHE_CH01947	K07708	ret:RHE_CH01948	K07712	100	100	OK
pfl:PFL_0378	K07708	pfl:PFL_0377	K07712	100	100	OK
dar:Daro_3759	K07708	dar:Daro_3758	K02481	100	100	bad KO (?)
dvu:DVU3382	K07709	dvu:DVU3381	K07713	50.2	23.6	OK
eba:ebA960	K07711	eba:ebA966	K02481	73.2	46.4	bad KO (?)
ppu:PP_5047	K07708	ppu:PP_5048	K07712	100	100	OK
sbo:SBO_4024	K07709	sbo:SBO_4025	K07713	74.8	50.6	OK
hch:HCH_06659	K07711	hch:HCH_06661	K07715	73.2	48.2	OK
eli:ELI_06280	K07708	eli:ELI_06275	K07712	100	100	OK
nmu:Nmul_A1159	K07711	nmu:Nmul_A2671	K02481	100	100	x
ece:Z5579	K07709	ece:Z5580	K07713	74.8	50.6	OK
atc:AGR_C_2663	K07708	atc:AGR_C_2665	K07712	100	100	OK
gme:Gmet_2562	K07708	gme:Gmet_2453	K02481	30	-40	bad KO (?)
hpy:HP0244	K07710	hpy:HP0703	K02481	100	100	OK
ypa:YPA_3519	K07708	ypa:YPA_3520	K07712	97.4	94.8	OK
cca:CCA00157	K07710	cca:CCA00155	K02481	100	100	OK
dde:Dde_0110	K07709	dde:Dde_0109	K07713	73	48.6	OK
abo:ABO_2260	K07708	abo:ABO_2259	K07712	100	100	OK
stm:STM2564	K07711	stm:STM2562	K07715	73.2	48	OK
bpa:BPP2987	K07708	bpa:BPP2986	K07712	100	100	OK
bja:blr4487	K07708	bja:blr4488	K07712	100	100	OK
spt:SPA0302	K07711	spt:SPA0304	K07715	73.2	48	OK
ecp:ECP_2558	K07711	ecp:ECP_2556	K07715	73.2	48	OK
she:Shewmr4_0258	K07708	she:Shewmr4_0257	K07712	100	100	OK
rme:Rmet_2060	K07708	rme:Rmet_2059	K07712	100	100	OK
rpa:RPA2592	K07708	rpa:RPA2593	K07712	100	100	OK
ecs:ECs4926	K07709	ecs:ECs4927	K07713	74.8	50.6	OK
cpj:CPj0584	K07710	cpj:CPj0586	K02481	100	100	OK

mag:amb2366	K07708	mag:amb2367	K07712	100	100	OK
sec:SC2559	K07711	sec:SC2557	K02481	73.2	48	bad KO (?)
sfl:SF3939	K07708	sfl:SF3938	K07712	97.4	94.8	OK
bmf:BAB1_1141	K07708	bmf:BAB1_1140	K07712	100	100	OK
ppr:PBPRA2770	K08475	ppr:PBPRA2771	K08476	100	100	OK
sdy:SDY_3874	K07708	sdy:SDY_3875	K07712	97.4	94.8	OK
ype:YPO0023	K07708	ype:YPO0022	K07712	97.4	94.8	OK
pfo:Pfl_0340	K07708	pfo:Pfl_0339	K07712	100	100	OK
eba:ebA4117	K07708	eba:ebA4116	K02481	100	100	bad KO (?)
ecs:ECs3422	K07711	ecs:ECs3420	K07715	73.2	48	OK
nwi:Nwi_1444	K07708	nwi:Nwi_1445	K07712	100	100	OK
cpt:CpB0608	K07710	cpt:CpB0610	K02481	100	100	OK
ypn:YPN_0250	K07708	ypn:YPN_0249	K07712	97.4	94.8	OK
ece:Z3833	K07711	ece:Z3830	K07715	73.2	48	OK
lip:LI1078	K07709	lip:LI1079	K07713	74.8	49.6	OK
sec:SC2398	K08475	sec:SC2397	K08476	100	100	OK
ecv:APECO1_4340	K07710	ecv:APECO1_4339	K07714	100	100	OK
eco:b4003	K07709	eco:b4004	K07713	74.8	50.6	OK
eca:ECA3257	K07711	eca:ECA3255	K07715	97.4	94.8	OK
sfx:S3807	K07708	sfx:S3808	K07712	97.4	94.8	OK
xac:XAC0207	K07708	xac:XAC0208	K07712	100	100	OK
yps:YPTB2876	K07711	yps:YPTB2874	K07715	73.2	48	OK
ecp:ECP_4080	K07708	ecp:ECP_4079	K07712	97.4	94.8	OK
ecv:APECO1_3975	K07711	ecv:APECO1_3977	K07715	73.2	48	OK
ecc:c4961	K07709	ecc:c4962	K07713	74.8	50.6	OK
bte:BTH_I1845	K07708	bte:BTH_I1846	K07712	100	100	OK
vfi:VF0096	K07708	vfi:VF0095	K07712	100	100	OK
pat:Patl_1027	K07708	pat:Patl_1028	K07712	97.4	94.8	OK
neu:NE0015	K07711	neu:NE0017	K07715	64.8	29.6	OK
cpa:CP0164	K07710	cpa:CP0162	K02481	100	100	OK
bbu:BB0764	K02482	bbu:BB0763	K02481	100	100	OK
ype:YPO2916	K07711	ype:YPO2914	K07715	97.4	94.8	OK
pol:Bpro_1119	K07711	pol:Bpro_1117	K02481	100	100	bad KO (?)
eco:b3869	K07708	eco:b3868	K07712	97.4	94.8	OK
spt:SPA4010	K07709	spt:SPA4011	K02481	74.8	50.6	bad KO (?)
pca:Pcar_0496	K07711	pca:Pcar_0498	K07715	100	100	OK
psp:PSPPH_4852	K07708	psp:PSPPH_4853	K07712	100	100	OK
cvi:CV3591	K07708	cvi:CV3592	K07712	100	100	OK
vpa:VP0119	K07708	vpa:VP0118	K07712	100	100	OK
ppr:PBPRA3494	K07708	ppr:PBPRA3495	K07712	100	100	OK
ypn:YPN_1222	K07711	ypn:YPN_1224	K07715	73.2	48	OK
reu:Reut_A2055	K07708	reu:Reut_A2054	K02481	100	100	bad KO (?)
wsu:WS1590	K02482	wsu:WS0367	K02481	100	100	OK
ypm:YP_0984	K07709	ypm:YP_0983	K07713	74.8	50.6	OK
ypm:YP_2541	K07711	ypm:YP_2543	K07715	73.2	48	OK
ssn:SSON_4176	K07709	ssn:SSON_4177	K07713	74.8	50.6	OK
sec:SC4054	K07709	sec:SC4055	K02481	74.8	50.6	bad KO (?)
vpa:VPA0826	K08475	vpa:VPA0827	K08476	100	100	OK
pae:PA5124	K07708	pae:PA5125	K07712	100	100	OK

ecp:ECP_2262	K07710	ecp:ECP_2263	K07714	100	100	OK
ecc:c4818	K07708	ecc:c4817	K07712	97.4	94.8	OK
nar:Saro_1927	K07708	nar:Saro_1928	K07712	100	100	OK
sec:SC3898	K07708	sec:SC3897	K02481	97.4	94.8	bad KO (?)
sdn:Sden_3457	K07708	sdn:Sden_3458	K07712	100	100	OK
tbd:Tbd_2501	K07708	tbd:Tbd_2500	K07712	100	100	OK
bbr:BB2953	K07708	bbr:BB2952	K07712	100	100	OK
stm:STM4173	K07709	stm:STM4174	K07713	74.8	50.6	OK
pha:PSHAa0620	K07711	pha:PSHAa0622	K07715	100	100	OK
ypa:YPA_2355	K07711	ypa:YPA_2353	K07715	73.2	48	OK
ecj:JW3840	K07708	ecj:JW3839	K07712	97.4	94.8	OK
son:SO4471	K07708	son:SO4472	K07712	100	100	OK
dar:Daro_1802	K07711	dar:Daro_1804	K02481	73	46	bad KO (?)
ssn:SSON_2278	K07710	ssn:SSON_2279	K07714	100	100	OK
sbo:SBO_2584	K07711	sbo:SBO_2582	K07715	73.2	48	OK
stt:t3615	K07708	stt:t3616	K07712	97.4	94.8	OK
aae:aq_231	K02482	aae:aq_230	K02481	75.4	50.8	OK
rfr:Rfer_2981	K07708	rfr:Rfer_2982	K07712	100	100	OK
xcb:XC_0197	K02482	xcb:XC_0198	K02481	100	100	OK
ecc:c5203	K08475	ecc:c5204	K08476	100	100	OK
cps:CPS_0398	K07708	cps:CPS_0397	K07712	100	100	OK
spt:SPA3847	K07708	spt:SPA0464	K08476	97.4	94.8	bad KO (?)
bpe:BP1597	K07708	bpe:BP1598	K07712	100	100	OK
tcx:Tcr_2189	K07711	tcx:Tcr_1444	K02481	100	100	x
cpn:CPn0584	K07710	cpn:CPn0586	K02481	100	100	OK
atu:Atu1445	K07708	atu:Atu1446	K07712	100	100	OK
ssn:SSON_4041	K07708	ssn:SSON_4040	K07712	97.4	94.8	OK
ecc:c2762	K07710	ecc:c2763	K07714	100	100	OK
cps:CPS_4270	K07711	cps:CPS_4272	K07715	100	100	OK
ypm:YP_0024	K07708	ypm:YP_0023	K07712	97.4	94.8	OK
aci:ACIAD1369	K07708	aci:ACIAD1368	K07712	100	100	OK
ypk:y1313	K07711	ypk:y1315	K07715	97.4	97.4	OK
eco:b2219	K07710	eco:b2220	K07714	100	100	OK
bmb:BruAb1_1124	K07708	bmb:BruAb1_1123	K02481	100	100	bad KO (?)
bme:BMEI0865	K07708	bme:BMEI0866	K07712	100	100	OK
sgl:SG2231	K07708	sgl:SG2232	K07712	97.4	94.8	OK
ece:Z5405	K07708	ece:Z5404	K07712	97.4	94.8	OK
vch:VC2748	K07708	vch:VC2749	K07712	100	100	OK
hhe:HH0140	K02482	hhe:HH1635	K02481	100	100	OK
ecj:JW3967	K07709	ecj:JW3968	K07713	74.8	50.6	OK
yps:YPTB0023	K07708	yps:YPTB0022	K07712	97.4	94.8	OK
hch:HCH_01429	K07711	hch:HCH_00696	K02481	94.6	89.2	x
sfl:SF2603	K07711	sfl:SF2601	K07715	73.2	48	OK
eci:UTI89_C2501	K07710	eci:UTI89_C2502	K07714	100	100	OK
rpd:RPD_2589	K07708	rpd:RPD_2590	K07712	100	100	OK
pca:Pcar_1993	K07708	pca:Pcar_1994	K07712	100	100	OK
rru:Rru_A1677	K07708	rru:Rru_A1678	K07712	100	100	OK
yps:YPTB1204	K07709	yps:YPTB1205	K07713	74.8	50.6	OK
gsu:GSU1004	K07708	gsu:GSU1320	K02481	29.6	-40.8	bad KO (?)

cfe:CF0850	K07710	cfe:CF0852	K02481	100	100	OK
psb:Psyr_4821	K07708	psb:Psyr_4822	K07712	100	100	OK
pha:PSHAa0164	K07708	pha:PSHAa0163	K07712	100	100	OK
eca:ECA0028	K07708	eca:ECA0027	K07712	97.4	94.8	OK
bga:BG0787	K02482	bga:BG0786	K02481	100	100	OK
bma:BMA1741	K07708	bma:BMA1740	K07712	100	100	OK
xfa:XF1849	K07708	xfa:XF1848	K07712	100	100	OK
hpj:jhp0229	K02482	hpj:jhp0643	K02481	100	100	OK
xcc:XCC0188	K07708	xcc:XCC0189	K07712	100	100	OK
ecv:APECO1_2593	K07708	ecv:APECO1_2594	K07712	97.4	94.8	OK
ctr:CT467	K07710	ctr:CT468	K02481	100	100	OK
sil:SPO2088	K02482	sil:SPO2087	K02481	100	100	OK
rso:RSc1260	K07708	rso:RSc1261	K02481	100	100	bad KO (?)
stt:t0461	K08475	stt:t0462	K08476	100	100	OK
xcv:XCV0191	K07708	xcv:XCV0192	K07712	100	100	OK
rpb:RPB_2883	K07708	rpb:RPB_2882	K07712	100	100	OK
ssn:SSON_2639	K07711	ssn:SSON_2637	K07715	73.2	48	OK
rfr:Rfer_2788	K07711	rfr:Rfer_2786	K02481	73.2	46.4	bad KO (?)
cmu:TC0752	K07710	cmu:TC0753	K02481	100	100	OK
vvy:VVA0716	K08475	vvy:VVA0717	K08476	100	100	OK
pmu:PM1459	K08475	pmu:PM1460	K08476	100	100	OK
stt:t0292	K07711	stt:t0293	K07715	73.2	48	OK
hch:HCH_01034	K07708	hch:HCH_01033	K07712	99.2	98.4	OK
bpm:BURPS1710b_2768	K07708	bpm:BURPS1710b_2766	K07712	100	100	OK
sty:STY2634	K08475	sty:STY2633	K08476	100	100	OK
sdy:SDY_3723	K07709	sdy:SDY_3722	K07713	74.8	50.6	OK
ecj:JW2213	K07710	ecj:JW2214	K07714	100	100	OK
pol:Bpro_1809	K07708	pol:Bpro_1810	K07712	100	100	OK
vch:VCA0705	K08475	vch:VCA0704	K08476	100	100	OK
eco:b2556	K07711	eco:b2554	K07715	73.2	48	OK
sme:SMc01042	K07708	sme:SMc01043	K07712	100	100	OK
mlo:mlr0397	K07708	mlo:mlr0398	K07712	100	100	OK
xft:PD1020	K07708	xft:PD1021	K07712	100	100	OK
par:Psyc_1537	K07708	par:Psyc_1538	K07712	100	100	OK
cvi:CV4218	K07711	cvi:CV4220	K07715	100	100	OK
sty:STY2811	K07711	sty:STY2809	K07715	73.2	48	OK
cta:CTA_0511	K07710	cta:CTA_0512	K02481	100	100	OK
ecv:APECO1_3508	K08475	ecv:APECO1_3507	K08476	100	100	OK
stt:t3458	K07709	stt:t3457	K07713	74.8	50.6	OK
vvy:VV0197	K07708	vvy:VV0196	K07712	100	100	OK
tbd:Tbd_2769	K07711	tbd:Tbd_2771	K07715	68	36	OK
eci:UTI89_C2876	K07711	eci:UTI89_C2873	K07715	73.2	71.6	OK
sty:STY3875	K07708	sty:STY3876	K07712	97.4	94.8	OK
aae:aq_1115	K02482	aae:aq_164	K02481	75.4	50.8	OK
sfx:S3660	K07709	sfx:S3659	K07713	74.8	50.6	OK
rsp:RSP_2837	K07708	rsp:RSP_2838	K02481	99.8	99.6	bad KO (?)
plu:plu3313	K07711	plu:plu3311	K07715	97.4	94.8	OK
vvu:VV2_0209	K08475	vvu:VV2_0210	K08476	100	100	OK
sty:STY3712	K07709	sty:STY3711	K07713	74.8	50.6	OK

bps:BPSL2317	K07708	bps:BPSL2316	K07712	100	100	OK
sfl:SF4075	K07709	sfl:SF4076	K07713	74.8	50.6	OK
shm:Shewmr7_3763	K07708	shm:Shewmr7_3764	K07712	100	100	OK
mca:MCA1715	K07711	mca:MCA1713	K07715	100	100	OK
bur:Bcep18194_A5457	K07708	bur:Bcep18194_A5456	K07712	100	100	OK
ecp:ECP_4216	K07709	ecp:ECP_4217	K07713	74.8	50.6	OK
stm:STM4006	K07708	stm:STM4005	K07712	97.4	94.8	OK
ecj:JW5407	K07711	ecj:JW2538	K07715	73.2	48	OK
vvu:VV1_0891	K07708	vvu:VV1_0892	K07712	100	100	OK
sfr:Sfri_0321	K07708	sfr:Sfri_0320	K07712	100	100	OK
sal:Sala_1276	K07708	sal:Sala_0950	K02481	97.2	94.4	x
sdy:SDY_2746	K07711	sdy:SDY_2744	K07715	73.2	48	OK
ecs:ECs4791	K07708	ecs:ECs4790	K07712	97.4	94.8	OK
sbo:SBO_3881	K07708	sbo:SBO_3880	K07712	97.4	94.8	OK
xoo:XOO4484	K07708	xoo:XOO4483	K02481	100	100	bad KO (?)
mfa:Mfla_2449	K07708	mfa:Mfla_2450	K02481	100	100	bad KO (?)
shn:Shewana3_0259	K07708	shn:Shewana3_0258	K07712	100	100	OK

Figure S2. Graphical representation of the predicted pairings between the sensor kinase and response regulator families of the Ntr-like two component system corresponding to the best mapping found by TAG-TSEMA. The colour of the links corresponds to their “reliability” score according to the scale shown at the bottom (see Izarzugaza *et al.* (2006). Nucleic Acids Res., 34:W315-319).

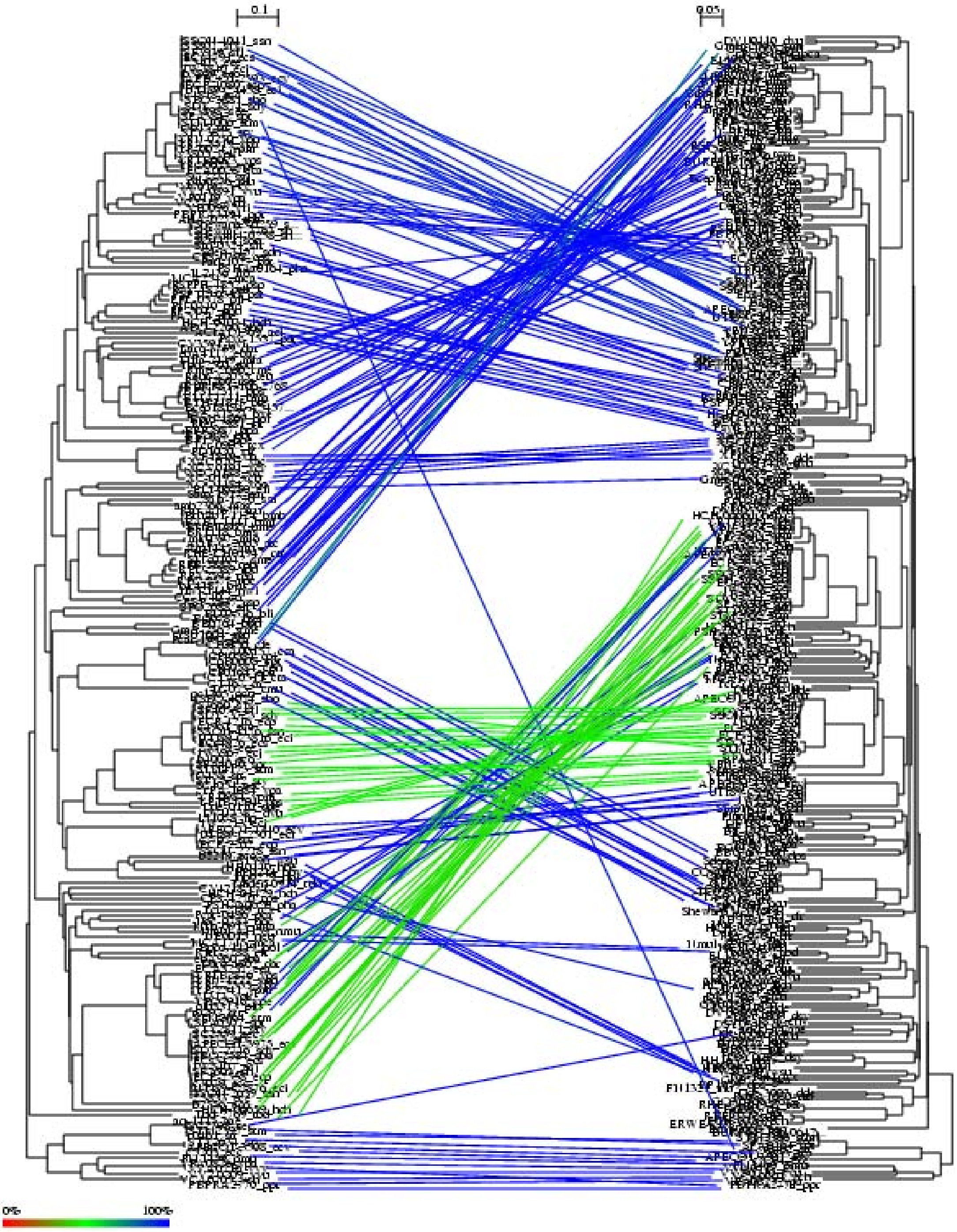

Figure S3. Relationship between the number of organisms, the average number of sequences per organism and the accuracy. The bottom-left panel shows the a 3D representation of the space defined by these three variables, while the other panels show different 2D projections of that space. The accuracy is also represented with a color gradient (right).

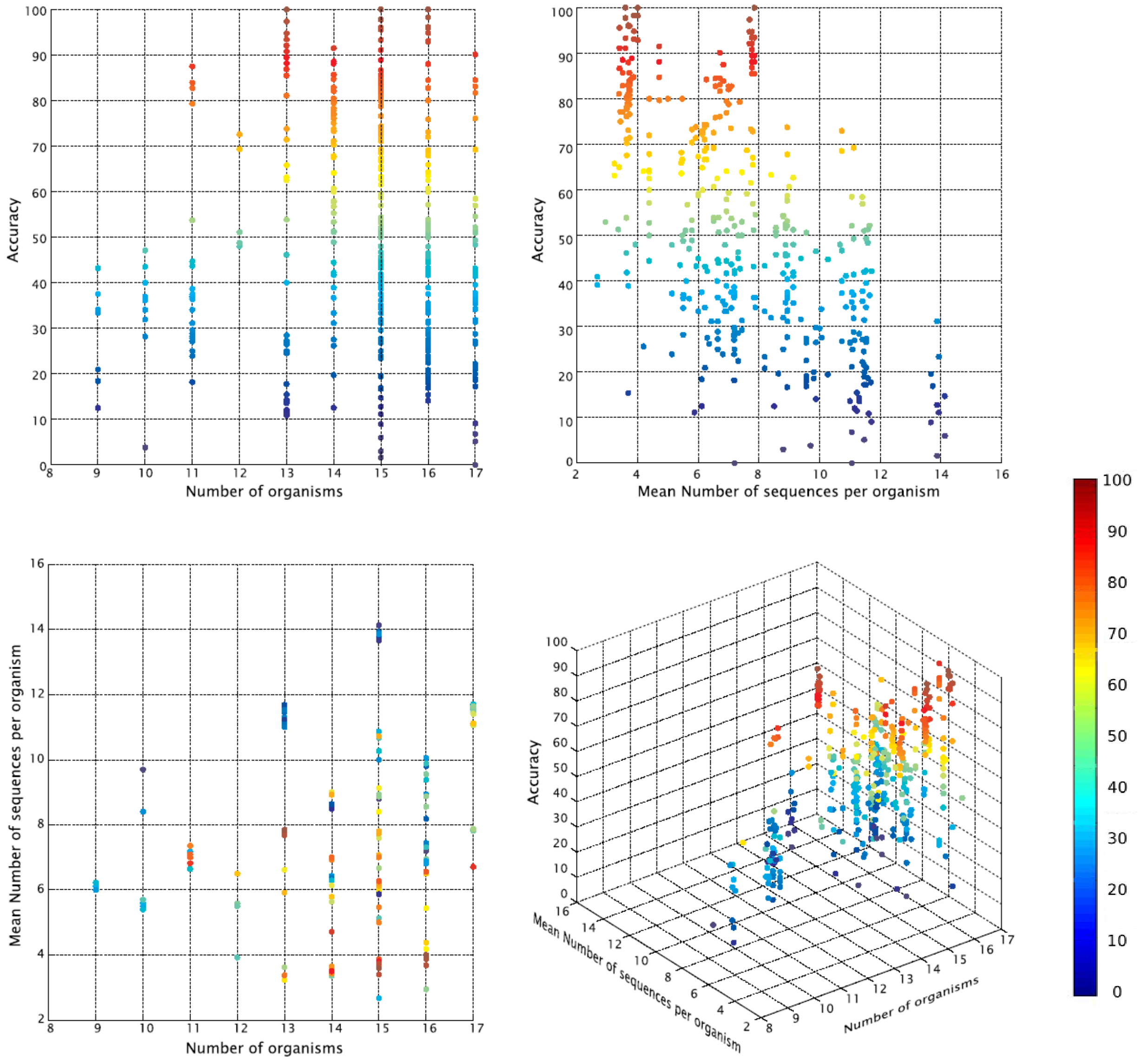

Supplement: Additional File 1 — Supplementary tables S1 and S2 and supplementary figures S1, S2 and S3 [file 1471-2105-9-35-S1.pdf]
